# Cathepsin-D, urokinase plasminogen activator and type-1 plasminogen activator inhibitor in early breast cancer: an immunohistochemical study of prognostic value and relations to tenascin-C and other factors

**DOI:** 10.1038/sj.bjc.6690336

**Published:** 1999-04

**Authors:** T Jahkola, T Toivonen, K von Smitten, I Virtanen, V-M Wasenius, C Blomqvist

**Affiliations:** Fourth Department of Surgery, Helsinki University Central Hospital, Kasarmikatu 11–13, FIN-00130 Helsinki, Finland; Department of Pathology, Kymenlaakso Central Hospital, FIN-48210 Kotka, Finland; Department of Anatomy, Institute of Biomedicine, FIN-00014 University of Helsinki, Finland; Department of Oncology, Helsinki University Central Hospital, FIN-00290 Helsinki, Finland

**Keywords:** cathepsin-D, uPA, PAI-1, immunohistochemistry, early breast cancer, breast-conserving surgery, metastasis, local recurrence

## Abstract

Cytosolic determinations of cathepsin-D (cath-D), urokinase plasminogen activator (uPA) and its specific inhibitor PAI-1 have shown an association with adverse prognosis in breast cancer. Our aim was to study the distribution of these markers in small axillary node-negative breast carcinomas using immunohistochemistry and relate the semiquantitative results to known prognostic factors, the expression of tenascin-C (Tn-C) in invasion border of the tumour and prognosis. All the 158 women (159 tumours) were treated with breast conserving surgery and postoperative radiotherapy. Cytoplasmic immunoreactivity for cath-D was seen in carcinoma cells in 47% and in stromal cells in 44%. Nearly all tumours expressed uPA and PAI-1, which were categorized to cytoplasmic expression in carcinoma cells and diffuse stromal expression and quantified – / + / ++ / +++ and further dichotomized for purposes of analysis. Expression of uPA and PAI-1 in stromal fibroblasts was recorded as – / +. Cytoplasmic and stromal cell cath-D contents were associated with grade, proliferation, Tn-C expression in the tumour invasion border and the development of distant metastasis. In multivariate analysis stromal cath-D proved to be an independent prognostic factor for metastasis. Stromal expression of uPA was associated with an increased risk of local recurrence; otherwise high levels of uPA did not associate with other prognostic factors nor with prognosis. Fibroblastic expression of PAI-1 showed an association with both local and distant disease recurrence. However, no consistent association between the immunohistochemically quantified uPA and PAI-1 and prognosis was found. In conclusion, immunohistochemical determination of cath-D seems to be a viable method to predict a higher risk of metastasis but not local recurrence in small axillary node-negative breast carcinomas.© 1999 Cancer Research Campaign


British Journal of Cancer (1999) 80(1/2), 167–174
© 1999 Cancer Research Campaign
Article no. bjoc.1998.0336

### Cathepsin-D, urokinase plasminogen activator and type-1 plasminogen activator inhibitor in early breast cancer: an immunohistochemical study of prognostic value and relations to tenascin-C and other factors

**T Jahkola[1], T Toivonen[2], K von Smitten[1], I Virtanen[3], V-M Wasenius[4] and C Blomqvist[4]**

[1]Fourth Department of Surgery, Helsinki University Central Hospital, Kasarmikatu 11–13, FIN-00130 Helsinki, Finland; [2]Department of Pathology, Kymenlaakso Central Hospital, FIN-48210 Kotka, Finland; [3]Department of Anatomy, Institute of Biomedicine, FIN-00014 University of Helsinki, Finland; [4]Department of Oncology, Helsinki University Central Hospital, FIN-00290 Helsinki, Finland

**Summary** Cytosolic determinations of cathepsin-D (cath-D), urokinase plasminogen activator (uPA) and its specific inhibitor PAI-1 have shown an association with adverse prognosis in breast cancer. Our aim was to study the distribution of these markers in small axillary node-negative breast carcinomas using immunohistochemistry and relate the semiquantitative results to known prognostic factors, the expression of tenascin-C (Tn-C) in invasion border of the tumour and prognosis. All the 158 women (159 tumours) were treated with breast conserving surgery and postoperative radiotherapy. Cytoplasmic immunoreactivity for cath-D was seen in carcinoma cells in 47% and in stromal cells in 44%. Nearly all tumours expressed uPA and PAI-1, which were categorized to cytoplasmic expression in carcinoma cells and diffuse stromal expression and quantified – / + / ++ / +++ and further dichotomized for purposes of analysis. Expression of uPA and PAI-1 in stromal fibroblasts was recorded as – / +. Cytoplasmic and stromal cell cath-D contents were associated with grade, proliferation, Tn-C expression in the tumour invasion border and the development of distant metastasis. In multivariate analysis stromal cath-D proved to be an independent prognostic factor for metastasis. Stromal expression of uPA was associated with an increased risk of local recurrence; otherwise high levels of uPA did not associate with other prognostic factors nor with prognosis. Fibroblastic expression of PAI-1 showed an association with both local and distant disease recurrence. However, no consistent association between the immunohistochemically quantified uPA and PAI-1 and prognosis was found. In conclusion, immunohistochemical determination of cath-D seems to be a viable method to predict a higher risk of metastasis but not local recurrence in small axillary node-negative breast carcinomas.

**Keywords:** cathepsin-D; uPA; PAI-1; immunohistochemistry; early breast cancer; breast-conserving surgery; metastasis; local recurrence

The concerted action of proteolytic enzymes that either promote or directly take part in extracellular matrix (ECM) and basement membrane (BM) degradation and remodelling is considered of main importance in cancer invasion and metastatic spread (Bernstein and Liotta, 1994). These proteinases are excreted by different cells like macrophages, fibroblasts, normal or malignant epithelial cells as inactive pro-enzymes, zymogens. Their function is regulated by other proteinases, growth-factors and specific inhibitors. Cathepsin D (cath-D) and urokinase plasminogen activator (uPA) are the two most widely studied proteolytic enzymes in breast cancer research.

Cathepsin D is an aspartic lysosomal proteinase that is active in acidic pH. Cell culture studies suggest that cath-D may be active in carcinomas either as a proteolytic enzyme or a mitogen (Westley and May, 1996). Since 1989 the independent adverse prognostic sign of high cath-D concentrations of tissue extracts in breast cancer has been demonstrated in numerous studies (Thorpe et al, 1989; Duffy et al, 1991, 1992; Kute et al, 1991; Namer et al, 1991; Pujol et al, 1993; Fernö et al, 1994), also showing prognostic power in node-negative patients (Spyratos et al, 1989; Tandon et al, 1990; Granata et al, 1991). However, studies using immunohistochemistry to detect cath-D in carcinoma cells have provided controversial results of cath-D as a prognostic factor in breast cancer. The different results may be due to differences in patient selection, follow-up times, antibodies or cut-off values (Ravdin, 1993; Cardiff, 1994; Rochefort, 1996; Westley and May, 1996). Three reports describe an adverse prognostic value for the expression of cath-D in stromal cells but not in tumour cells (Tetû et al, 1993; Joensuu et al, 1995; O'Donoghue et al, 1995).

Urokinase plasminogen activator (uPA) is a serine protease, which activates zymogen forms of other proteases like plasmin and gelatinase A (also called MMP-2 or type IV collagenase) and these both degrade type IV collagen (Liotta et al, 1981; Dano et al, 1985; Keski-Oja et al, 1992), one of the main constituents of the basement membranes. Type 1 plasminogen activator inhibitor (PAI-1) is a specific inhibitor of uPA and its expression is enhanced in malignant breast tissue along with uPA as compared with normal breast tissue (Foucré et al, 1991; Reilly et al, 1992; Costantini et al, 1996). Several studies have demonstrated that increasing amounts or activity of uPA and PAI-1 are associated with aggressive growth properties and worse prognosis in breast cancer. These results have often been independent of other prognostic factors leading to the suggestion that high levels of proteolytic enzymes indicate a more aggressive disease within a defined

*Received 16 June 1998*
*Revised 26 August 1998*
*Accepted 11 September 1998*

*Correspondence to:* T Jahkola

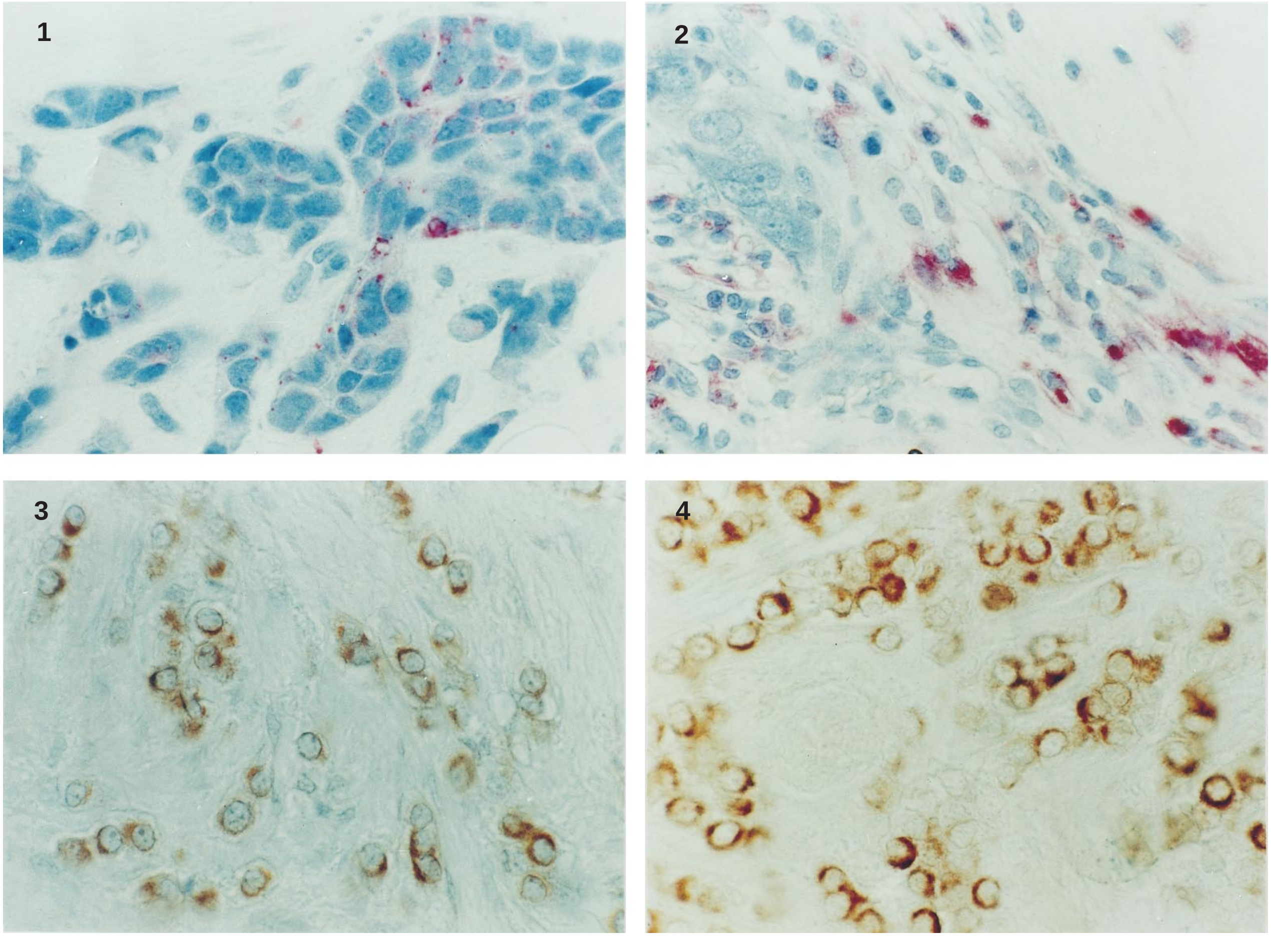

**Figures 1–4** Positive immunoreactivity for cathepsin-D in carcinoma cells (Figure 1) and in stromal cells (Figure 2) in ductal breast carcinomas. Positive immunoreactivity for uPA (Figure 3) and PAI-1 (Figure 4) in the cytoplasm of lobular breast carcinoma patient group and thus could be of use to select patients for adjuvant therapies (Duffy et al, 1988; Jänicke et al, 1989, 1993; Foekens et al, 1992, 1994; Spyratos et al, 1992; Grondal-Hansen et al, 1993, 1997; Bouchet et al, 1994). High contents of uPA and PAI-1 in renal carcinoma tissue appear to be strong and independent prognostic factors (Hofmann et al, 1996). The high-grade immunohistochemical uPA staining of epithelial cells in Dukes' B colorectal cancer has also been discovered to be of prognostic value (Mulcahy et al, 1994).

Immunohistochemistry uses only one 4–6 μm slice of formalin-fixed tissue for each analysis and saves tissue for other diagnostic and prognostic studies. This is a clear advantage in small tumours. Our purpose was to study the distribution and level of the immunoreactivities for cath-D, u-PA and PAI-1 and their possible prognostic value to predict local or distant recurrence in axillary node-negative small breast carcinomas all treated with breast saving surgery. The archival material has been characterized earlier and several prognostic factors have been evaluated. The previously studied prognostic factors include tumour size, histology, grade, possible intraductal component (DCIS), ploidy, proliferation measured with S phase fraction (SPF) and Ki-67 antigen expression, erbB-2 oncoprotein, p53 protein and tenascin-C (Tn-C) expression (Jahkola et al, 1998). The expression of the extracellular matrix glycoprotein Tn-C in the invasion border of these very same tumours is a prognostic factor for both local recurrence after breast saving surgery and distant metastasis (Jahkola et al, 1998). The prognostic significances of cath-D, uPA and PAI-1 immunoreactivities, and their relationships with other prognostic factors, are analysed here.

### PATIENTS AND METHODS

### Patients

All patients included in this study had undergone breast saving surgery and post-operative radiotherapy (25 × 2 Gy) but no adjuvant hormonal- or chemotherapy for axillary node-negative invasive breast carcinoma. The patients and histopathological characteristics of the tumours have been described in detail earlier (Jahkola et al, 1998). In short, the original patient group of 143 women (144 tumours, one bilateral) was followed for a median of 7.8 years (range 5.4–11.4 years) during which seven local recurrences (5%) and 14 distant metastases (10%) were diagnosed four patients having both. Due to the small number of recurrences a new additional patient group was included to strengthen the statistical power of the study. This additional group consisted of 15 women with a recurrent disease (seven local recurrences and 11 distant metastases three having both) selected from a patient population

 © Cancer Research Campaign 1999

**Table 1** Associations between cath-D, uPA and PAI-1 in their different areas of expression in small axillary node-negative breast carcinomas

| | All tumours | Cytoplasmic cath-D positive (%) | Stromal cell cath-D positive (%) | Cytoplasmic uPA high grade (%) | Stromal uPA high grade (%) | Fibroblastic uPA positive (%) | Cytoplasmic PAI-1 high grade (%) | Stromal PAI-1 high grade (%) | Fibroblastic PAI-1 positive (%) |
|---|---|---|---|---|---|---|---|---|---|
| All tumours | 159 | 73 (47) | 68 (44) | 96 (63) | 70 (46) | 44 (29) | 60 (40) | 50 (33) | 47 (31) |
| Cytoplasmic | | | | | | | | | |
| cath-D+ | 73 | – | | | | | | | |
| cath-D– | 82 | – | | | | | | | |
| Stromal cell | | | | | | | | | |
| cath-D+ | 68 | 43 (63) | – | | | | | | |
| cath-D– | 87 | 30 (34) | – | | | | | | |
| | | *P* = 0.0004*** | | | | | | | |
| Cytoplasmic | | | | | | | | | |
| uPA high grade | 96 | 47 (49) | 46 (48) | – | | | | | |
| uPA low grade | 57 | 24 (44) | 18 (33) | – | | | | | |
| | | *P* = 0.5 | *P* = 0.06 | | | | | | |
| Stromal | | | | | | | | | |
| uPA high grade | 70 | 28 (41) | 26 (38) | 47 (67) | – | | | | |
| uPA low grade | 83 | 43 (53) | 38 (47) | 49 (59) | – | | | | |
| | | *P* = 0.1 | *P* = 0.3 | *P* = 0.3 | | | | | |
| Fibroblastic | | | | | | | | | |
| uPA+ | 44 | 21 (48) | 27 (61) | 38 (86) | 25 (57) | – | | | |
| uPA– | 109 | 50 (47) | 37 (35) | 58 (53) | 45 (41) | – | | | |
| | | *P* = 0.9 | *P* = 0.003* | *P* < 0.0001*** | *P* = 0.08 | | | | |
| Cytoplasmic | | | | | | | | | |
| PAI-1 high grade | 60 | 32 (54) | 25 (42) | 45 (76) | 24 (41) | 18 (31) | – | | |
| PAI-1 low grade | 91 | 38 (43) | 39 (44) | 48 (53) | 45 (50) | 26 (29) | – | | |
| | | *P* = 0.2 | *P* = 0.9 | *P* = 0.005** | *P* = 0.3 | *P* = 0.8 | | | |
| Stromal | | | | | | | | | |
| PAI-1 high grade | 50 | 27 (55) | 18 (37) | 27 (54) | 27 (54) | 16 (32) | 28 (56) | – | |
| PAI-1 low grade | 101 | 41 (43) | 45 (47) | 66 (68) | 42 (42) | 28 (28) | 32 (32) | – | |
| | | *P* = 0.2 | *P* = 0.2 | *P* = 0.09 | *P* = 0.2 | *P* = 0.6 | *P* = 0.004* | | |
| Fibroblastic | | | | | | | | | |
| PAI-1+ | 47 | 27 (57) | 29 (62) | 43 (93) | 15 (33) | 22 (48) | 26 (55) | 14 (30) | – |
| PAI-1– | 104 | 43 (43) | 35 (35) | 50 (49) | 54 (52) | 22 (21) | 34 (33) | 36 (35) | – |
| | | *P* = 0.09 | *P* = 0.002** | *P* < 0.0001*** | *P* = 0.03* | *P* = 0.001*** | *P* = 0.01* | *P* = 0.6 | – |

with similar treatment for node-negative breast carcinoma and equal recurrence rates as the original patient group (Jahkola et al, 1998).

### Immunohistochemical analysis of cath-D, uPA and PAI-1

Formalin-fixed and paraffin-embedded tumour samples were cut to 4-μm-thick sections, deparaffinized and endogenous peroxidase was blocked for 30 min in 0.3% hydrogen peroxide in methanol. Alkaline phosphatase anti-alkaline phosphatase (APAAP kit system 40, cat. no. K0670, DAKO A/S, Denmark) and streptavidin–peroxidase (StreptAB complex/HRP kit, cat. no. K492, DAKO A/S, Denmark) methods were applied according to the manufacturer's instructions. 3-Amino-9-ethyl carbazole was used as chromogen for peroxidase, Mayer's haematoxylin was used for nuclear staining and the sections were mounted with Aquamount (BDH Ltd, Poole, UK). Mouse monoclonal antibodies against cath-D (clone 1C11, Triton Diagnostics, Alameda, CA, USA), uPA (cat. No 3689, American Diagnostica Inc., Greenwich, CT, USA) and PAI-1 (cat. No. 3785, American Diagnostica Inc., Greenwich, CT, USA) were applied at dilutions of 1:20 (0.13 $\mu g\ ml^{-1}$, 1 h at room temperature), 1:75 (13 $\mu g\ ml^{-1}$, 1 h at 37°C) and 1:100 (10 $\mu g\ ml^{-1}$, 1 h at 37°C) respectively. For cath-D, liver sections were used as positive controls, and for uPA and PAI-1, positive controls were breast carcinoma specimens known to stain for the markers. Negative controls consisted of incubations with omission of primary antibodies.

All tumours were evaluated for cath-D, uPA and PAI-1 expression by a pathologist unaware of the outcome of patients. A few samples in each set of immunohistochemistry could not be interpreted due to technical failures or lack of carcinoma tissue.

The expression of cath-D was typically granular and confined to cytoplasm of carcinoma cells, but also of stromal macrophages and fibroblasts. We noticed no preference of location of cath-D-positive cells between central parts and periphery of the tumours. The scoring of cytoplasmic cath-D immunoreactivity was assessed according to Isola et al (1993): tumours with a clearly detectable level of 10% or more of strongly positive carcinoma cells were defined as positive for cytoplasmic reactivity. When 10% or more of stromal cells were strongly positive, the stromal expression was called positive respectively (Tetû et al, 1993) (Figures 1 and 2).

Immunoreactivity for uPA and PAI-1 were seen in a vast majority of tumours. Expression was seen in the cytoplasm of carcinoma cells (Figures 3 and 4) and diffusely in the surrounding stroma, but also frequently in the cytoplasm of benign breast epithelia and stroma (not shown). For uPA and PAI-1, the classifi-

*© Cancer Research Campaign 1999*

**Table 2** Associations of cath-D, uPA and PAI-1 with histopathological and prognostic factors in small axillary node-negative breast carcinomas

| | All tumours | Cytoplasmic cath-D positive (%) | Stromal cell cath-D positive (%) | Cytoplasmic uPA high grade (%) | Stromal uPA high grade (%) | Fibroblastic uPA positive (%) | Cytoplasmic PAI-1 high grade (%) | Stromal PAI-1 high grade (%) | Fibroblastic PAI-1 positive (%) |
|---|---|---|---|---|---|---|---|---|---|
| All tumours | 159 | 73 (47) | 68 (44) | 96 (63) | 70 (46) | 44 (29) | 60 (40) | 50 (33) | 47 (31) |
| Histology | | | | | | | | | |
| Ductal | 95 | 47 (51) | 44 (47) | 54 (59) | 40 (44) | 28 (29) | 35 (38) | 36 (40) | 33 (35) |
| Lobular | 35 | 16 (47) | 12 (35) | 24 (67) | 17 (49) | 7 (20) | 17 (49) | 9 (26) | 9 (26) |
| Others[a] | 29 | 10 (36) | 12 (43) | 18 (67) | 13 (48) | 9 (31) | 8 (33) | 5 (22) | 5 (17) |
| | | $P = 0.4$ | $P = 0.5$ | $P = 0.6$ | $P = 0.9$ | $P = 0.5$ | $P = 0.4$ | $P = 0.1$ | $P = 0.2$ |
| Grade[b] | | | | | | | | | |
| 1 | 51 | 12 (24) | 20 (40) | 28 (61) | 19 (41) | 14 (27) | 14 (30) | 20 (43) | 12 (24) |
| 2 | 34 | 21 (62) | 12 (35) | 22 (67) | 15 (45) | 10 (29) | 15 (45) | 11 (34) | 14 (41) |
| 3 | 26 | 16 (67) | 17 (71) | 12 (46) | 13 (50) | 9 (35) | 9 (35) | 9 (34) | 9 (35) |
| | | $P = 0.0002$*** | $P = 0.02$* | $P = 0.3$ | $P = 0.8$ | $P = 0.8$ | $P = 0.4$ | $P = 0.6$ | $P = 0.2$ |
| DCIS+[c] | 57 | 28 (50) | 25 (47) | 32 (58) | 27 (49) | 14 (25) | 19 (35) | 26 (48) | 15 (26) |
| DCIS– | 102 | 45 (45) | 43 (43) | 64 (65) | 43 (44) | 30 (29) | 41 (43) | 24 (25) | 32 (31) |
| | | $P = 0.6$ | $P = 0.9$ | $P = 0.4$ | $P = 0.5$ | $P = 0.5$ | $P = 0.3$ | $P = 0.005$** | $P = 0.5$ |
| EIC+[d] | 12 | 8 (67) | 6 (50) | 6 (55) | 6 (55) | 2 (17) | 5 (45) | 4 (36) | 3 (25) |
| EIC– | 147 | 65 (45) | 62 (43) | 90 (63) | 64 (45) | 42 (29) | 55 (39) | 46 (34) | 44 (30) |
| | | $P = 0.2$ | $P = 0.7$ | $P = 0.6$ | $P = 0.5$ | $P = 0.4$ | $P = 0.7$ | $P = 0.9$ | $P = 0.7$ |
| erbB-2+[e] | 11 | 4 (36) | 3 (27) | 5 (45) | 7 (64) | 3 (27) | 5 (45) | 7 (64) | 1 (9) |
| erbB-2– | 131 | 58 (45) | 54 (42) | 80 (62) | 55 (43) | 37 (28) | 52 (42) | 37 (30) | 35 (27) |
| | | $P = 0.6$ | $P = 0.3$ | $P = 0.3$ | $P = 0.2$ | $P = 0.9$ | $P = 0.8$ | $P = 0.02$* | $P = 0.2$ |
| p53 +[f] | 22 | 13 (59) | 13 (59) | 11 (55) | 9 (45) | 7 (32) | 4 (19) | 6 (30) | 8 (36) |
| p53 – | 121 | 53 (45) | 52 (44) | 79 (68) | 55 (47) | 34 (28) | 51 (44) | 40 (35) | 36 (30) |
| | | $P = 0.2$ | $P = 0.2$ | $P = 0.3$ | $P = 0.9$ | $P = 0.7$ | $P = 0.03$* | $P = 0.6$ | $P = 0.5$ |
| Ki-67+[g] | 80 | 45 (58) | 48 (62) | 52 (68) | 32 (42) | 28 (35) | 29 (37) | 29 (38) | 28 (35) |
| Ki-67– | 61 | 20 (33) | 16 (26) | 39 (67) | 29 (50) | 15 (25) | 28 (49) | 17 (30) | 17 (28) |
| | | $P = 0.003$** | $P < 0.0001$*** | $P = 0.97$ | $P = 0.3$ | $P = 0.18$ | $P = 0.2$ | $P = 0.4$ | $P = 0.4$ |
| SPF high[h] | 73 | 38 (53) | 39 (54) | 45 (64) | 33 (47) | 25 (35) | 25 (35) | 20 (29) | 24 (33) |
| SPF low | 72 | 28 (39) | 22 (31) | 42 (61) | 28 (41) | 15 (20) | 28 (42) | 22 (34) | 20 (27) |
| | | $P = 0.1$ | $P = 0.005$** | $P = 0.7$ | $P = 0.4$ | $P = 0.05$* | $P = 0.4$ | $P = 0.5$ | $P = 0.4$ |
| Aneuploid | 55 | 30 (55) | 28 (51) | 30 (57) | 20 (38) | 23 (42) | 23 (43) | 25 (47) | 19 (35) |
| Diploid | 95 | 39 (42) | 37 (40) | 60 (66) | 44 (48) | 18 (19) | 32 (36) | 21 (24) | 25 (26) |
| | | $P = 0.1$ | $P = 0.2$ | $P = 0.3$ | $P = 0.2$ | $P = 0.003$** | $P = 0.4$ | $P = 0.006$** | $P = 0.3$ |

[a]others: two papillary, one medullary, two mucinous, 14 tubular, ten tubulolobular; [b]grade of 111 ductal and tubular carcinomas; [c]DCIS = intraductal component; [d]EIC = extensive intraductal component; [e]erbB-2 stained only of the original patient material of 143; [f]p53 cut-off 20% of nuclei positive; [g]Ki-67 cut-off 5% of nuclei positive; [h]SPF cut-offs: median SPF of diploid tumours 2.4% and median of aneuploid tumours 8.4%.

cation of cytoplasmic and diffuse stromal immunoreactivities was performed visually taking into account both intensity and the proportion of cells or area of the tumour stained. The categories were negative (–), slightly (+), moderately (++), or strongly, positive (+++). In some samples the stromal fibroblasts showed a moderate to strong intensity, which phenomenon seemed independent of the diffuse stromal immunoreactivity and this was recorded separately (–/+). For purposes of analysis the categories of cytoplasmic and diffuse stromal uPA and PAI-1 reactions were combined to form a low-grade and a high-grade group for both cytoplasmic and diffuse stromal staining of uPA and PAI-1 (see Results for distribution).

### Statistical methods

Chi-square test, Fisher's exact test and Mann–Whitney *U*-test were used to test for association between variables. The statistical significance of differences in outcome between patients with or without a prognostic factor was calculated using the Cox proportional hazard model to compute the hazard ratios (HR) of disease recurrence. The statistical significance of the effect of the continuous variables on disease recurrence was also tested with the Cox proportional hazard model with the variable to be tested as the only covariate. The multivariate analysis for metastasis was performed with the Cox proportional hazard model entering the variables significant in the univariate analysis. All the tests were two-sided and *P*-values smaller than 0.05 were considered significant.

### RESULTS

### The distribution of immunoreactivity

In 155 out of 159 tumours cath-D expression could be interpreted. There were 73 tumours (47%) showing immunoreactivity for cytoplasmic cath-D and 68 tumours (44%) for stromal cell expression. Cross-tabulation of cytoplasmic and stromal cell cath-D reactivities showed an association ($P = 0.0004$) with 43 tumours (28%) expressing both (Table 1).

Staining for uPA could be interpreted in 153 tumours and PAI-1 in 151 tumours. Cytoplasmic uPA was missing in nine tumours

**Table 3** Association of tenascin-C expression in invasion border with cytoplasmic and stromal expression of cath-D, uPA and PAI-1 in axillary node-negative breast carcinomas

| | All tumours | Tn-C positive invasion border (%) | Chi-square *P*-value |
|---|---|---|---|
| All | 121 | 63 (52) | |
| Cytoplasmic | | | |
| cath-D+ | 53 | 33 (62) | 0.05 |
| cath-D– | 66 | 29 (44) | |
| Stromal cell | | | |
| cath-D+ | 54 | 34 (63) | 0.03 |
| cath-D– | 65 | 28 (43) | |
| Cytoplasmic | | | |
| uPA high grade | 75 | 43 (57) | 0.3 |
| uPA … low grade | 41 | 19 (46) | |
| Stromal | | | |
| uPA … high grade | 54 | 31 (57) | 0.3 |
| uPA … low grade | 65 | 31 (48) | |
| Fibroblastic | | | |
| uPA+ | 39 | 24 (62) | 0.15 |
| uPA– | 82 | 39 (48) | |
| Cytoplasmic | | | |
| PAI-1 high grade | 45 | 22 (48) | 0.5 |
| PAI-1 … low grade | 69 | 40 (55) | |
| Stromal | | | |
| PAI-1 high grade | 35 | 21 (60) | 0.3 |
| PAI-1 low grade | 84 | 41 (49) | |
| Fibroblastic | | | |
| PAI-1+ | 42 | 27 (64) | 0.05 |
| PAI-1– | 79 | 36 (46) | |

An invasion border was present in 121 tumours out of the 159 in the archival specimens available. Cath-D cut-off 10% of cells strongly positive, for cut-offs of uPA and PAI-1 see text.

(6%), + in 48 tumours (31%), ++ in 73 tumours (48%) and +++ in 23 tumours (15%). For analyses groups, – and + were combined to form a low-grade group of 57 tumours (37%), and ++ and +++ to form a high-grade group of 96 tumours (63%). Stromal uPA was noted in all tumours. In 83 (54%) it was weak and this was called low-grade in the analyses. Moderate expression was seen in 59 tumours (39%) and strong in 11 tumours (7%) and these were combined to one high-grade stromal uPA group of 70 tumours (46%).

Cytoplasmic PAI-1 was expressed in all tumours. In 19 (12%) it was weak, in 72 (48%) moderate and in 60 (40%) strong. For analyses, groups + and ++ were combined to present a low-grade cytoplasmic PAI-1 group of 90 tumours (60%). Stromal PAI-1 was missing in 21 tumours (13%), + in 80 tumours (53%), ++ in 48 tumours (32%) and +++ in two tumours (1%). For analyses, – and + were combined to one low-grade stromal PAI-1 group of 101 tumours (67%), and ++ and +++ to a high-grade group of 50 tumours (33%).

### Relations within proteolytic enzymes

High levels of stromal cell cath-D was associated with cytoplasmic cath-D and expression of uPA and PAI-1 in fibroblasts, but not with cytoplasmic or diffuse stromal expressions of uPA and PAI-1. Generally the cytoplasmic expression of uPA and PAI-1 associated with each other and with fibroblastic expressions. The diffuse stromal uPA associated only with fibroblastic PAI-1. The stromal content of PAI-1 showed an association with cytoplasmic and fibroblastic PAI-1, but not with uPA or cath-D (Table 1).

### Relations with other prognostic factors

Tumour size, histology, grade according to Bloom and Richardson, the immunohistochemical detection of erbB-2 oncoprotein, p53 protein and Ki-67 antigen, as well as the flow-cytometric measurement of ploidy and SPF have been performed and presented earlier (Jahkola et al, 1998). There were no significant differences in the distributions of cath-D, uPA and PAI-1 with respect to patient age or tumour size (data not shown).

Cytoplasmic cath-D expression was associated with grade ($P$ = 0.0002) and proliferation measured with Ki-67 ($P$ = 0.003) (Table 2). Cath-D expression in stromal cells was associated also with grade ($P$ = 0.02) and proliferation ($P$ < 0.0001 for Ki-67 and $P$ = 0.005 for SPF) (Table 2, cut-offs in footnotes).

There was no association between stromal or cytoplasmic uPA and other prognostic factors listed in Table 2. Fibroblastic uPA showed an association with high SPF ($P$ = 0.05) and aneuploidy ($P$ = 0.003) (Table 2).

Cytoplasmic PAI-1 associated with low or missing p53 expression ($P$ = 0.02). Stromal expression of PAI-1 was associated with intraductal component (DCIS) ($P$ = 0.005), erbB-2 ($P$ = 0.02) and aneuploidy ($P$ = 0.006), whereas fibroblastic PAI-1 expression did not show association with any of the listed prognostic factors (Table 2).

### Relations with the expression of Tn-C in invasion border

An invasion border could be identified in 121 out of the 159 tumours in the available archival specimens. The expression of Tn-C at the invasive front of these tumours was shown earlier to correlate to higher risk of distant metastasis (Jahkola et al, 1996, 1998*a*) and local recurrence after breast-conserving surgery (Jahkola et al, 1998*a*). A Tn-C-positive invasion border was associated with cath-D expression both in carcinoma and stromal cells. There was also an association between Tn-C and fibroblastic PAI-1 (Table 3).

### Univariate analysis for distant and local recurrence

In univariate analysis for metastasis, cytoplasmic cath-D (HR = 3.80, confidence intervals (CI) = 1.50–9.57, $P$ = 0.005), stromal cell cath-D (HR = 4.28, CI = 1.70–10.78, $P$ = 0.002) and fibroblastic PAI-1 (HR = 2.44, CI = 1.06–5.61, $P$ = 0.04) were significant prognostic factors. Our immunohistochemical quantification failed to show any association between uPA and metastasis. For local recurrence, only stromal uPA (HR = 7.24, CI = 1.62–32.34, $P$ = 0.01) and fibroblastic PAI-1 (HR = 4.3, CI = 1.44–12.85, $P$ = 0.009) showed prognostic power (Table 4).

The previously shown prognostic factors for metastasis are size (HR = 2.56, CI = 1.13–5.94, $P$ = 0.02), SPF of diploid tumours (HR = 3.16, CI = 1.02–9.81, $P$ = 0.05), Ki-67 (HR = 5.5, CI = 1.6–18.7, $P$ = 0.006) and Tn-C in the invasion border (HR = 3.4, CI = 1.1–10.4, $P$ = 0.03). For local recurrence only Tn-C in the invasion border was significant in predicting local recurrence (HR = 11.0, CI = 1.4–85.1, $P$ = 0.02).

© Cancer Research Campaign 1999

**Table 4** Hazard ratios (HR) for local recurrence and metastasis in 158 women with 159 axillary node-negative breast carcinomas treated with breast-saving surgery and post-operative radiotherapy

| | HR for local recurrence | *P*-value | HR for metastasis | *P*-value |
|---|---|---|---|---|
| Cytoplasmic | | | | |
| cath-D+ | 1.63 | 0.37 | 3.8 | 0.005 |
| cath-D– | 1.0 | | 1.0 | |
| Stromal | | | | |
| cath-D+ | 2.4 | 0.12 | 4.28 | 0.002 |
| cath-D– | 1.0 | | 1.0 | |
| Cytoplasmic | | | | |
| uPA high grade | 2.22 | 0.22 | 1.51 | 0.37 |
| uPA low grade | 1.0 | | 1.0 | |
| Stromal | | | | |
| uPA high grade | 7.24 | 0.01 | 1.03 | 0.94 |
| uPA low grade | 1.0 | | 1.0 | |
| Fibroblastic | | | | |
| uPA+ | 1.01 | 0.92 | 0.88 | 0.88 |
| uPA– | 1.0 | | 1.0 | |
| Cytoplasmic | | | | |
| PAI-1 high grade | 0.62 | 0.42 | 1.23 | 0.62 |
| PAI-1 low grade | 1.0 | | 1.0 | |
| Stromal | | | | |
| PAI-1 high grade | 0.82 | 0.74 | 1.08 | 0.86 |
| PAI-1 low grade | 1.0 | | 1.0 | |
| Fibroblastic | | | | |
| PAI-1+ | 4.31 | 0.009 | 2.44 | 0.04 |
| PAI-1– | 1.0 | | 1.0 | |

Cath-D cut-off 10% of cells strongly positive, for cut-offs of uPA and PAI-1 see text.

**Table 5** Multivariate analysis (Cox proportional hazard model) of the three covariates statistically significant in univariate analysis of metastasis

| Covariate | *P* | HR | CI (95%) |
|---|---|---|---|
| Cytoplasmic cath-D | 0.068 | 2.46 | 0.94–6.45 |
| Stromal cell cath-D | 0.034 | 2.88 | 1.08–7.71 |
| Fibroblastic PAI-1 | 0.24 | 1.66 | 0.71–3.89 |

**Table 6** Multivariate analysis (Cox proportional hazard model) of the two covariates statistically significant in univariate analysis of local recurrence

| Covariate | *P* | HR | CI (95%) |
|---|---|---|---|
| Diffuse stromal uPA | 0.001 | 8.06 | 2.64–24.65 |
| Fibroblastic PAI-1 | 0.0003 | 12.67 | 2.76–58.12 |

### Multivariate analysis for metastasis and local recurrence

When cytoplasmic and stromal cath-D and fibroblastic PAI-1 were introduced into the multivariate analysis, stromal cell cath-D remained the strongest prognostic factor for metastasis (Table 5). After adding the previously found prognostic factors for metastasis, Ki-67, Tn-C in the invasion border and tumour size, each at a time, Ki-67 remained as the strongest prognostic factor followed by stromal cath-D, Tn-C, cytoplasmic cath-D, fibroblastic PAI-1 and tumour size as the weakest (data not shown).

Both fibroblastic PAI-1 and stromal uPA remained statistically significant in the multivariate analysis for local recurrence (Table 6). When the previously studied Tn-C in invasion border was included, it turned out to be the weakest of these three (data not shown).

### DISCUSSION

This study showed a significant prognostic value for cath-D expression in carcinoma cells, but also in stromal cells, to predict a higher risk of distant metastasis. In the study of Henry et al (1990), cath-D expression in malignant epithelial cells was unexpectedly protective of disease recurrence in axillary node-positive patients whereas several other studies have not proven a statistically significant relation between cath-D expression and prognosis (Domagala et al, 1992; Kandalaft et al, 1993; Winstanley et al, 1993; Armas et al, 1994; Castiglioni et al, 1994; Ravdin et al, 1994; Alo et al, 1996). Using the same monoclonal antibody as in the present study, Isola et al (1993) found that the expression of cath-D in tumour cells was an independent and strong indicator of adverse prognosis in axillary node-negative patients ($n$ = 262). Another study correlated an intense cath-D immunostaining with worse prognosis in node-positive ($n$ = 76) but not in node-negative ($n$ = 66) patients (Aaltonen et al, 1995).

Three reports describe a statistically significant adverse prognostic value for the expression of cath-D in stromal cells but not tumour cells (Tetû et al, 1993; Joensuu et al, 1995; O'Donoghue et al, 1995). Tetû et al (1993) studied only axillary node-positive patients, and in the studies of Joensuu et al (1995) and O'Donoghue et al (1995) the number of axillary node-negative patients was relatively low (53 and 61 respectively). The cytosolic cath-D content has been correlated to carcinoma cells (Roger et al, 1994) and to stromal macrophages (Razumovic et al, 1997) suggesting that the cytosolic cath-D is the cumulative result of cath-D content in both carcinoma cells and stromal cells. Considering that the cytosolic studies have provided lots of evidence of the prognostic usefulness of cath-D in breast cancer, we conclude that cytoplasmic and stromal cath-D immunoreactivity can be used either alone or combined to predict the risk of systemic metastasis in early breast carcinoma patients. This is in accordance with the recent meta-analysis result of a relationship between high cath-D values and poor disease-free survival in node-negative breast cancer patients (Ferrandina et al, 1997).

The associations of cath-D with grade and proliferation have been reported earlier by others (Charpin et al, 1993; Joensuu et al, 1995). Unlike us, Isola et al found no association between cytoplasmic cath-D and grade (Isola et al, 1993). Stromal cell cath-D has been shown to scatter to the invasive area of the tumour (O'Donoghue et al, 1995). We did not notice this in the small tumours of our study. Instead, we found a significant correlation between both cytoplasmic and stromal cell content of cath-D and the expression of Tn-C in the invasion border that we suggest is related to invasion (Jahkola et al, 1998*b*).

Concerning uPA and PAI-1, our aim was to quantify immunohistochemically their contents in small node-negative breast carcinomas and relate the results to prognosis. We found a correlation between fibroblastic PAI-1 and both local and distant recurrence, and between diffuse stromal uPA and local recurrence. There were also correlations between cytoplasmic and fibroblastic expression of uPA and PAI-1, indicating perhaps a general increase in proteolytic

 *© Cancer Research Campaign 1999*

activity in carcinomas. This has previously been suggested in studies comparing the immunohistochemical expressions of uPA and PAI-1 in normal breast epithelia, in benign tumours and in carcinomas (Reilly et al, 1992; Bianchi et al, 1995; Christensen et al, 1996; Costantini et al, 1996). Christensen et al (1996) have described fibroblastic uPA and PAI-1 staining in a subgroup of poorly differentiated tumours which is in accordance with our result of a connection between fibroblastic PAI-1 and worse prognosis.

We failed to observe a consistent correlation between increased immunohistochemical levels of uPA and PAI-1 and prognosis. This may be due to the nearly ubiquitous expression of these components in normal breast tissue and difficulties in quantification thereof. Immunohistochemistry can only be semiquantitative, though anatomically accurate, and it seems that it cannot replace cytosolic measurements of uPA and PAI-1 for prognostic purposes. The immunohistochemical analysis for urokinase plasminogen activator receptor (uPAR) may be more useful since its presence is required for assembling an active uPA system at the surface of the cell, whereas the expression of uPA and PAI-1 at a certain localization may be either the site of production or the site of activity (Christensen et al, 1996).

In conclusion, our results show that the immunohistochemical analysis for cath-D in the cytoplasm of both carcinoma cells and stromal cells was related to increased risk of distant metastasis but not of local recurrence after breast-conserving surgery. In multivariate setting the expression in stromal cells was the strongest prognostic factor for metastasis. Cath-D immunoreactivity was associated with grade and proliferation as well as with the expression of Tn-C in tumour invasion border. We failed to produce a consistent immunohistochemical quantification of uPA and PAI-1 for prognostic purposes. Fibroblastic PAI-1 expression associated with both local recurrence and distant metastasis, and diffuse stromal uPA associated with local recurrence. These studies have to be repeated in the future before definitive conclusions can be drawn.

### ACKNOWLEDGEMENTS

The technical assistance of Ms L Pirjamo, K Messina, M-L Piironen and Mr R Karppinen is acknowledged. We thank Dr J Keski-Oja for review of the manuscript. This study was supported by the Finnish Cancer Organization and the Kurt and Doris Palander's Foundation.

### REFERENCES

Aaltonen M, Lipponen P, Kosma V-M, Aaltomaa S and Syrjänen K (1995) Prognostic value of cathepsin D expression in female breast cancer. *Anticancer Res* **15**: 1033–1038

Alo PL, Visca P, Marci A, Mangoni A, Botti C and Tondo UD (1996) Expression of fatty acid synthase (FAS) as a predictor of recurrence in stage I breast carcinoma patients. *Cancer* **77**: 474–482

Armas OA, Gerald WL, Lesser ML, Arroyo CD, Norton L and Rosen PP (1994) Immunohistochemical detection of cathepsin D in T2N0M0 breast carcinoma. *Am J Surg Pathol* **18**: 158–166

Bernstein LR and Liotta LA (1994) Molecular mediators of interactions with extracellular matrix components in metastasis and angiogenesis. *Curr Opin Oncol* **6**: 106–113

Bianchi E, Cohen RL, Dai A, Thor AT, Shuman MA and Smith HS (1995) Immunohistochemical localization of the plasminogen activator inhibitor-1 in breast cancer. *Int J Cancer* **60**: 597–603

Bouchet C, Spyratos F, Martin PM, Hacène K, Gentile A and Oglobine J (1994) Prognostic value of urokinase-type plasminogen activator (uPA) and plasminogen activator inhibitors PAI-1 and PAI-2 in breast carcinomas. *Br J Cancer* **69**: 398–405

Cardiff RD (1994) Cathepsin D and breast cancer: useful? (editorial). *Hum Pathol* **25**: 847–848

Castiglioni T, Merino MJ, Elsner B, Lah TT, Sloane BF and Emmert-Buck MR (1994) Immunohistochemical analysis of cathepsins D, B, and L in human breast cancer. *Hum Pathol* **25**: 857–862

Charpin C, Devictor B, Bonnier P, Andrac L, Lavaut MN, Allasia C and Piana L (1993) Cathepsin D immunocytochemical assays in breast carcinomas: image analysis and correlation to prognostic factors. *J Pathol* **170**: 463–470

Christensen L, Simonsen ACW, Heegaard CW, Moestrup SK, Andersen JA and Andreasen PA (1996) Immunohistochemical localization of urokinase-type plasminogen activator, type-1 plasminogen activator inhibitor, urokinase receptor and alfa-2-macroglobulin receptor in human breast carcinomas. *Int J Cancer* **66**: 441–452

Costantini V, Sidoni A, Deveglia R, Cazzato OA, Bellezza G, Ferri I, Bucciarelli E and Nenci GG (1996) Combined overexpression of urokinase, urokinase receptor, and plasminogen activator inhibitor-1 is associated with breast cancer progression. *Cancer* **77**: 1079–1088

Dano K, Andreasen PA, Grondal-Hansen J, Kristensen P, Nielsen LS and Skriver L (1985) Plasminogen activators, tissue degradation and cancer. *Adv Cancer Res* **44**: 139–266

Domagala W, Striker G, Szadowska A, Dukowicz A, Weber K and Osborn M (1992) Cathepsin D in invasive ductal NOS breast carcinoma as defined by immunohistochemistry. *Am J Pathol* **141**: 1003–1012

Duffy MJ, O'Grady P, Devaney D, O'Siorain L, Fennelly JJ and Lijnen HJ (1988) Urokinase-plasminogen activator, a marker for aggressive breast carcinomas: preliminary report. *Cancer* **62**: 531–533

Duffy MJ, Reilly D, O'Sullivan C, O'Higgins N, Fennelly JJ and Andreasen P (1990) Urokinase-plasminogen activator, a new and independent prognostic marker in breast cancer. *Cancer Res* **50**: 6827–6829

Duffy MJ, Brouillet J-P, Reilly D, McDermott E, O'Higgins N, Fennelly JJ, Maudelonde T and Rochefort H (1991) Cathepsin D concentration in breast cancer cytosols: correlation with biochemical, histological, and clinical findings. *Clin Chem* **37**: 101–104

Duffy MJ, Reilly D, Brouillet J-P, McDermott EWM, Faul C, O'Higgins N, Fennelly JJ, Maudelonde T and Rochefort H (1992) Cathepsin D concentration in breast cancer cytosols: correlation with disease-free interval and overall survival. *Clin Chem* **38**: 2114–2116

Fernö M, Baldetorp B, Borg Å, Brouillet J-P, Olsson H, Rochefort H, Sellberg G, Sigurdsson H, Killander D and Group t.S.S.B.C. (1994) Cathepsin D, both a prognostic factor and a predictive factor for the effect of adjuvant tamoxifen in breast cancer. *Eur J Cancer* **30A**: 2042–2048

Ferrandina G, Scambia G, Bardelli F, Panici PB, Mancuso S and Messori A (1997) Relationship between cathepsin-D content and disease-free survival in node-negative breast cancer patients: a meta-analysis. *Br J Cancer* **76**: 661–666

Foekens JA, Schmitt M, von Putten WLJ, Peters HA, Bontenbal M, Jänicke F and Klijn JGM (1992) Prognostic value of urokinase-type plasminogen activator in 671 primary breast cancer patients. *Cancer Res* **52**: 6101–6105

Foekens JA, Schmitt M, von Putten WLJ, Peters HA, Kramer MD, Jänicke F and Klijn JGM (1994) Plasminogen activator inhibitor-1 and prognosis in primary breast cancer. *J Clin Oncol* **12**: 1648–1658

Foucré D, Bouchet C, Hacène K, Pourreau-Schneider N, Gentile A, Martin PM, Desplaces A and Oglobine J (1991) Relationship between cathepsin D, urokinase, and plasminogen activator inhibitors in malignant vs benign breast tumours. *Br J Cancer* **64**: 926–932

Granata G, Coradini D, Cappelletti V and Fronzo GD (1991) Prognostic relevance of cathepsin D versus oestrogen receptors in node negative breast cancers. *Eur J Cancer* **27**: 970–972

Grondal-Hansen J, Christensen IJ, Rosenquist C, Brunner N, Mouridsen HT, Dano K and Blichert-Toft M (1993) High levels of urokinase-type plasminogen activator and its inhibitor PAI-1 in cytosolic extracts of breast carcinomas are associated with poor prognosis. *Cancer Res* **53**: 2513–2521

Grondal-Hansen J, Hilsenbeck SG, Christensen IJ, Clark GM, Osborne CK and Brunner N (1997) Prognostic significance of PAI-1 and uPA in cytosolic extracts obtained from node-positive breast cancer patients. *Breast Cancer Res Treat* **43**: 153–163

Henry JA, McCarthy AL, Angus B, Westley BR, May FEB, Nicholson S, Cairns J, Harris AL and Horne CHW (1990) Prognostic significance of the estrogen-regulated protein, cathepsin D, in breast cancer. *Cancer* **65**: 265–271

Hofmann R, Lehmer A, Buresch M, Hartung R and Ulm K (1996) Clinical relevance of urokinase plasminogen activator, its receptor, and its inhibitor in patients with renal cell carcinoma. *Cancer* **78**: 487–492

Isola J, Weitz S, Visakorpi T, Holli K, Shea R, Khabbaz N and Kallioniemi O-P (1993) Cathepsin D expression detected by immunohistochemistry has

© Cancer Research Campaign 1999 

independent prognostic value in axillary node-negative breast cancer. *J Clin Oncol* **11**: 36–43

Jahkola T, Toivonen T, von Smitten K, Blomqvist C and Virtanen I (1996) Expression of tenascin in invasion border of early breast cancer correlates with higher risk of distant metastasis. *Int J Cancer (Pred Oncol)* **69**: 445–447

Jahkola T, Toivonen T, Virtanen I, von Smitten K, Nordling S, von Boguslawski K, Haglund C, Nevanlinna H and Blomqvist C (1998*a*) Tenascin-C expression in invasion border of early breast cancer – a predictor of local and distant recurrence. *Br J Cancer* **78**: 1507–1513

Jahkola T, Toivonen T, Nordling S, von Smitten K and Virtanen I (1998*b*) Expression of tenascin-C in intraductal carcinoma of human breast: relation to invasion. *Eur J Cancer* **34**: 1687–1692

Jänicke F, Schmitt M, Pache L, Ulm K, Harbeck N, Höfler H and Graeff H (1993) Urokinase (uPA) and its inhibitor PAI-1 are strong and independent prognostic factors in node-negative breast cancer. *Breast Cancer Res Treat* **24**: 195–208

Jänicke F, Schmitt M, Ulm K, Gössner W and Graeff H (1989) Urokinase-type plasminogen activator antigen and early relapse in breast cancer. *Lancet* **334**: 1049

Joensuu H, Toikkanen S and Isola J (1995) Stromal cell cathepsin D expression and long-term survival in breast cancer. *Br J Cancer* **71**: 155–159

Kandalaft PL, Chang KL, Ahn CW, Traweek ST, Mehta P and Battifora H (1993) Prognostic significance of immunohistochemical analysis of cathepsin D in low-stage breast cancer. *Cancer* **71**: 2756–2763

Keski-Oja J, Lohi J, Tuuttila A, Tryggvason K and Vartio T (1992) Proteolytic processing of the 72,000-Da type IV collagenase by urokinase plasminogen activator. *Exp Cell Res* **202**: 471–476

Kute TE, Shao ZM, Sugg NK, Long RT, Rusell GB and Case LD (1991) Cathepsin D as a prognostic indicator for node-negative breast cancer patients using both immunoassays and enzymatic assays. *Cancer Res* **52**: 5198–5203

Liotta LA, Tryggvason K, Garbisa S, Robey PG and Abe S (1981) Partial purification and characterization of a neutral protease which cleaves type IV collagen. *Biochemistry* **20**: 100–104

Mulcahy HE, Duffy MJ, Gibbons D, McCarthy P, Parfrey NA, O'Donoghue DP and Sheahan K (1994) Urokinase-type plasminogen activator and outcome in Dukes' B colorectal cancer. *Lancet* **344**: 583–584

Namer M, Ramaioli A, Fontana X, Etienne M-C, Héry M, Jourlait A, Milano G, Frenay M, Francois E and Lapalus F (1991) Prognostic value of total cathepsin D in breast tumors. *Breast Cancer Res Treat* **19**: 85–93

O'Donoghue AEMA, Poller DN, Bell JA, Galea MH, Elston CW, Blamey RW and Ellis IO (1995) Cathepsin D in primary breast carcinoma: adverse prognosis is associated with expression of cathepsin D in stromal cells. *Breast Cancer Res Treat* **33**: 137–145

Pujol P, Maudelonde T, Daures J-P, Rouanet P, Brouillet J-P, Pujol H and Rochefort H (1993) A prospective study of the prognostic value of cathepsin D levels in breast cancer cytosol. *Cancer* **71**: 2006–2012

Ravdin PM (1993) Evaluation of cathepsin D as a prognostic factor in breast cancer. (review). *Breast Cancer Res Treat* **24**: 219–226

Ravdin PM, Tandon AK, Allred C, Clark GM, Fuqua SAW, Hilsenbeck SH, Chamness GC and Osborne CK (1994) Cathepsin D by Western blotting and immunohistochemistry: failure to confirm correlations with prognosis in node-negative breast cancer. *J Clin Oncol* **12**: 467–474

Razumovic JJ, Stojkovic RR, Petrovecki M and Gamulin S (1997) Correlation of two methods for determination of cathepsin D in breast carcinoma (immunohistochemistry and ELISA in cytosol). *Breast Cancer Res Treat* **43**: 117–122

Reilly D, Christensen L, Duch M, Nolan N, Duffy MJ and Andreasen PA (1992) Type-1 plasminogen activator inhibitor in human breast carcinomas. *Int J Cancer* **50**: 208–214

Rochefort H (1996) The prognostic value of cathepsin D in breast cancer. A long road to the clinic. (editorial). *Eur J Cancer* **32A**: 7–8

Roger P, Montcourrier P, Maudelonde T, Brouillet J-P, Pages A, Laffargue F and Rochefort H (1994) Cathepsin D immunostaining in paraffin-embedded breast cancer cells and macrophages: correlation with cytosolic assay. *Hum Pathol* **25**: 863–871

Spyratos F, Brouillet J-P, Defrenne A, Hacene K, Rouessé J, Maudelonde T, Brunet M, Andrieu C, Desplaces A and Rochefort H (1989) Cathepsin D: an independent prognostic factor for metastasis of breast cancer. *Lancet* **334**: 1115–1118

Spyratos F, Martin P-M, Hacène K, Romain S, Andrieu C, Ferrero-Pous M, Deytieux S, Doussal VL, Tubiana-Hulin M and Brunet M (1992) Multiparametric prognostic evaluation of biological factors in primary breast cancer. *J Natl Cancer Inst* **84**: 1266–1272

Tandon AK, Clark GM, Chamness GC, Chirgwin JM and McGuire WL (1990) Cathepsin D and prognosis in breast cancer. *N Engl J Med* **322**: 297–302

Tetû B, Brisson J, Côté C, Brisson S, Potvin D and Roberge N (1993) Prognostic significance of cathepsin D expression in node-positive breast carcinoma: an immunohistochemical study. *Int J Cancer* **55**: 429–435

Thorpe SM, Rochefort H, Garcia M, Freiss G, Christensen IJ, Khalaf S, Paolucci F, Pau B, Rasmussen BB and Rose C (1989) Association between high concentrations of Mr 52,000 cathepsin D and poor prognosis in primary human breast cancer. *Cancer Res* **49**: 6008–6014

Westley BR and May FEB (1996) Cathepsin D and breast cancer. *Eur J Cancer* **32A**: 15–24

Winstanley JHR, Leinster SJ, Cooke TG, Westley BR, Platt-Higgins AM and Rudland PS (1993) Prognostic significance of cathepsin-D in patients with breast cancer. *Br J Cancer* **67**: 767–772

*© Cancer Research Campaign 1999*
